# Specific Risk Factors for Fatal Outcome in Critically Ill COVID-19 Patients: Results from a European Multicenter Study

**DOI:** 10.3390/jcm10173855

**Published:** 2021-08-27

**Authors:** David Meintrup, Stefan Borgmann, Karlheinz Seidl, Melanie Stecher, Carolin E. M. Jakob, Lisa Pilgram, Christoph D. Spinner, Siegbert Rieg, Nora Isberner, Martin Hower, Maria Vehreschild, Siri Göpel, Frank Hanses, Martina Nowak-Machen

**Affiliations:** 1 Faculty of Engineering and Management, University of Applied Sciences Ingolstadt, 85049 Ingolstadt, Germany; 2 Department of Infectious Diseases and Infection Control, Ingolstadt Hospital, 85049 Ingolstadt, Germany; stefan.borgmann@klinikum-ingolstadt.de; 3 Department of Cardiology, Ingolstadt Hospital, 85049 Ingolstadt, Germany; karlheinz.seidl@klinikum-ingolstadt.de; 4 Department I for Internal Medicine, University Hospital of Cologne, University of Cologne, 50937 Cologne, Germany; melanie.stecher@uk-koeln.de (M.S.); carolin.jakob2@uk-koeln.de (C.E.M.J.); 5 German Center for Infection Research (DZIF), Partner-Site Bonn-Cologne, 50937 Cologne, Germany; 6 Department of Internal Medicine, Hematology and Oncology, Goethe University Frankfurt, 60323 Frankfurt, Germany; lisa.pilgram@leoss.net; 7 Department of Internal Medicine II, University Hospital Rechts Der Isar, School of Medicine, Technical University of Munich, 81675 Munich, Germany; christoph.spinner@mri.tum.de; 8 German Center for Infection Research (DZIF), 38106 Brunswick, Germany; 9 Department of Medicine II, University of Freiburg, 79106 Freiburg, Germany; siegbert.rieg@uniklinik-freiburg.de; 10 Division of Infectious Diseases, Department of Internal Medicine II, University Hospital Würzburg, 97080 Würzburg, Germany; isberner_n@ukw.de; 11 Department of Pneumology, Infectious Diseases, Internal Medicine and Intensive Care, Klinikum Dortmund gGmbH, 44137 Dortmund, Germany; martin.hower@klinikumdo.de; 12 Department of Infectious Diseases, University Hospital Frankfurt, 60590 Frankfurt, Germany; maria.vehreschild@kgu.de; 13 Department of Internal Medicine I, University Hospital Tübingen, 72076 Tübingen, Germany; siri.goepel@med.uni-tuebingen.de; 14 German Center for Infection Research (DZIF), Clinical Research Unit for Healthcare Associated Infections, 72076 Tübingen, Germany; 15 Emergency Department and Department for Infection Control and Infectious Diseases, University Hospital Regensburg, 93053 Regensburg, Germany; frank.hanses@ukr.de; 16 Department of Anaesthesia and Intensive Care Medicine, Ingolstadt Hospital, 85049 Ingolstadt, Germany; martina.nowak-machen@klinikum-ingolstadt.de; 17 Department of Anesthesiology and Intensive Care Medicine, Teaching Faculty, University Hospital Tübingen, Eberhard-Karls-University, 72076 Tübingen, Germany

**Keywords:** COVID-19, SARS-CoV-2, risk factors, critically ill patients, comorbidities, lasso regression, nomogram

## Abstract

(1) Background: The aim of our study was to identify specific risk factors for fatal outcome in critically ill COVID-19 patients. (2) Methods: Our data set consisted of 840 patients enclosed in the LEOSS registry. Using lasso regression for variable selection, a multifactorial logistic regression model was fitted to the response variable survival. Specific risk factors and their odds ratios were derived. A nomogram was developed as a graphical representation of the model. (3) Results: 14 variables were identified as independent factors contributing to the risk of death for critically ill COVID-19 patients: age (OR 1.08, CI 1.06–1.10), cardiovascular disease (OR 1.64, CI 1.06–2.55), pulmonary disease (OR 1.87, CI 1.16–3.03), baseline Statin treatment (0.54, CI 0.33–0.87), oxygen saturation (unit = 1%, OR 0.94, CI 0.92–0.96), leukocytes (unit 1000/μL, OR 1.04, CI 1.01–1.07), lymphocytes (unit 100/μL, OR 0.96, CI 0.94–0.99), platelets (unit 100,000/μL, OR 0.70, CI 0.62–0.80), procalcitonin (unit ng/mL, OR 1.11, CI 1.05–1.18), kidney failure (OR 1.68, CI 1.05–2.70), congestive heart failure (OR 2.62, CI 1.11–6.21), severe liver failure (OR 4.93, CI 1.94–12.52), and a quick SOFA score of 3 (OR 1.78, CI 1.14–2.78). The nomogram graphically displays the importance of these 14 factors for mortality. (4) Conclusions: There are risk factors that are specific to the subpopulation of critically ill COVID-19 patients.

## 1. Introduction

The coronavirus disease 2019 (COVID-19) is caused by an infection with the novel coronavirus SARS-CoV-2. Starting in December 2019 in Wuhan, China, it quickly spread all over the world and turned into a pandemic. In June 2021, 18 months after the onset, more than 170 million incidences and more than 3.8 million COVID-19 related deaths were registered by health authorities worldwide [1].

From the very beginning of the pandemic, a large body of research focussed on identifying risk factors associated with COVID-19. As early as in February 2020, the Chinese Center for Disease Control published a first study on characteristics of COVID-19 patients [2]. By the time of writing in June 2021, a *Pubmed* search for articles containing “*COVID-19*” and “*risk factors*” in their title yielded more than 900 manuscripts.

Besides the search for factors generally associated with COVID-19, some authors focused on a specific comorbidity [3,4,5,6,7,8]. Others concentrate on demographic factors such as gender [9], ethnicity [10], or BMI [11]. A third branch of research invests risk factors for specific groups: pregnant women [12], the elderly population [13,14], or specific regions [15,16,17], to name a few examples. While in the beginning, identifying risk factors was of primary interest, there are now also some attempts to understand possible mechanisms [18,19,20]. Unsurprisingly, the vast amount of research has led to an equally high number of systematic reviews and meta-analyses on risk factors [21,22,23,24,25,26,27,28,29,30,31,32,33].

In developed countries, virtually all deaths from COVID-19 occur in nursing homes for the elderly, or in hospitals. While high age and a generally poor health condition associated with high age are known to be critical factors for residents of nursing homes, the analysis of hospitalized patients deserves special attention. Ethical standards for the admission to an intensive care unit (ICU) in case of limited resources have been published [34]. Numerous efforts have been made to predict the occurrence of critical illness for COVID-19 patients [35], typically at an early stage of the disease [36]. However, studies describing specific medical risk factors for already critically ill patients suffering from severe COVID-19 and a corresponding risk score are lacking to date.

In the present study, we sought to identify risk factors associated with death from COVID-19 for the subpopulation of critically ill hospitalized patients. Although these patients are under constant medical observation and typically treated on intensive care units, a high percentage of them succumb to the disease. In order to improve the outcome of COVID-19 patients on ICUs, it is critical to gain a better understanding of the specific risks of these patients.

Risk factors are typically identified by first applying a variable selection method based on univariate analysis or stepwise procedures. In a high-dimensional setting, both approaches are defective, and more sophisticated methods are required [37]. We used a multifactorial penalized logistic regression as variable selection tool to identify independent risk factors [38]. We were particularly interested to find out the similarities and differences between the risk factors of our patient sample and the risk factors for the general population known from the literature.

Finally, for the clinicians treating COVID-19 at ICU stations, an easy and fast assessment of the patient’s risk of death can be a valuable tool to decide about further treatment. As a first step, we developed a nomogram, a graphical representation of the model. It depicts the relative importance of each risk factor—demographics, clinical parameters and some common laboratory findings—for mortality.

## 2. Materials and Methods

### 2.1. Data Collection and Patients

At the beginning of the pandemic in March 2020, the Lean European Open Survey on SARS-CoV-2-Infected Patients (LEOSS) was started to collect data for epidemiological and clinical studies. The LEOSS project is a network of 146 active sites in 7 different European countries providing data of SARS-CoV-2 infected patients. LEOSS represents a non-interventional multi-center cohort study. Approval for LEOSS was obtained by the applicable local ethics committees of all participating centers, in particular the ethical committee of the Bavarian General Medical Council (project code 2020-1064) responsible for Ingolstadt hospital. The study was registered at the German Clinical Trials Register (DRKS, No. S00021145). More information on LEOSS can be found on the project’s website (https://leoss.net, accessed on 21 July 2021).

By July 16th 2021, data of 9755 cases were submitted to LEOSS. In LEOSS, patients are categorized in four clinical phases, which can roughly be characterized by asymptomatic/mild symptoms (uncomplicated phase), need for oxygen supplementation (complicated phase), need for critical care (critical phase), and the recovery phase. A detailed description of the critical phase is given below, and details for all phases can be found in Jakob et al. [39].

Patients are added to the registry retrospectively, once the treatment is completed or the patient died. In order to ensure anonymity in all steps of the analysis process, an individual LEOSS Scientific Use File (SUF) was created, which is based on the LEOSS Public Use File (PUF) principles described in Jakob et al. [40]. In particular, there is no need for informed consent.

This retrospective, observational study consists of 840 patients enclosed in the LEOSS registry between 23 March and 12 October 2020. All patients in the LOESS registry have a laboratory-confirmed SARS-CoV-2 infection, typically (94% of our patient sample) proven by at least one positive PCR test. Patients were included in our study if their SARS-CoV-2 infection entered the critical phase of the disease. The onset of the critical phase was declared if at least one of the following criteria was present: need for catecholamines, life-threatening cardiac arrhythmia, need for unplanned mechanical ventilation (invasive or non-invasive), prolongation (more than 24 h) of planned mechanical ventilation, liver failure with Quick <50% or INR >3.5, a quick SOFA score of two or higher, or acute renal failure in need of dialysis. A total of 101 patients were excluded because their last known status was neither recovered nor dead from COVID-19. Eighty-six percent of the critically ill patients included in our study were treated on ICUs for at least one day. In total, 78 (mostly German) medical centers contributed to the patient population under study.

We performed several steps to prepare the data set for statistical analysis. First, we excluded non-informative factors and factors with other data quality issues, e.g., a too high percentage of missing values, or an inconclusive coding scheme. From the remaining factors, we created 8 variables indicating presence of comorbidities. These 8 factors represent groups of diseases that were formed according to Table 1.

At that point, the data set contained 42 variables. Sixteen factors were recorded at baseline level: age, gender, BMI (body mass index), a known colonization with multi-resistant bacteria (MRB), four previous treatments (Angiotensin-converting enzyme (ACE) and angiotensin II type 1 (AT1) inhibitors, statins, immunosuppressive drugs), and the eight comorbidity factors. The 26 remaining factors were all measured during the critical phase of the COVID-19 disease. They included vital parameters (6), blood values (8), symptoms (8), and observations from CT scans (4) and are displayed in Table 2. It should be noted that for the vital parameters and blood values, the LEOSS database contains the worst measured value in the corresponding phase. This is a consequence of the anonymization design of the LEOSS registry, which only allows one to record one value per disease phase. In particular, this implicates that the values might not have been measured on the same day.

Originally, all variables were categorized and hence measured on a nominal or ordinal scale. In order to gain statistical power for the modeling process, we recoded age, BMI, the vital parameters and the blood values to continuous scale by using interval midpoints. Finally, we used boosted trees [41] including all other factors for data imputation. The final data set consisted of 739 patients, 1 binary response variable (recovered/dead), 16 baseline factors, and 26 factors recorded during the critical phase of the disease.

### 2.2. Statistical Analysis

Categorical variables are reported with absolute or relative frequencies. In order to identify risk factors associated with death of critically ill COVID-19 patients, we fitted a multifactorial logistic regression. Variables included in this analysis were the 16 baseline factors and the 26 factors listed in Table 2.

Due to the high total number of 42 factors, a variable selection mechanism was required prior to fitting the logistic model. As variable selection based on univariate analysis is defective [37,42], we used an L1-penalized adaptive model as multivariate tool for variable selection [38]. This procedure is also known as lasso (Least Absolute Shrinkage and Selection Operator) regression, and it has the additional advantage of taking care of multicollinearities among the factors. The lasso regression used the corrected Akaike Information Criterion (AICc) as validation method. As emphasis is on variable selection, the smallest model within a margin of 10 points of the minimal AICc was chosen. The factors selected by the lasso regression were then used to fit the final logistic regression. We then estimated odds ratios (with 95% confidence intervals) for independent significant factors in the multifactorial model. These odds ratios can be used to assess the independent contribution of the corresponding factor to the risk of death. All these statistical analyses were performed with the statistical software JMP${}^{®}$ Pro [43].

In order to generate a graphical representation of the risk factors we built a nomogram based on the results of the multivariate logistic regression. The nomogram was constructed using the rms package in R (version 4.0.4, R Foundation for Statistical Computing; http://www.r-project.org/ (accessed on 30 June 2021).

## 3. Results

### 3.1. Descriptive Statistics

**Figure 1:**
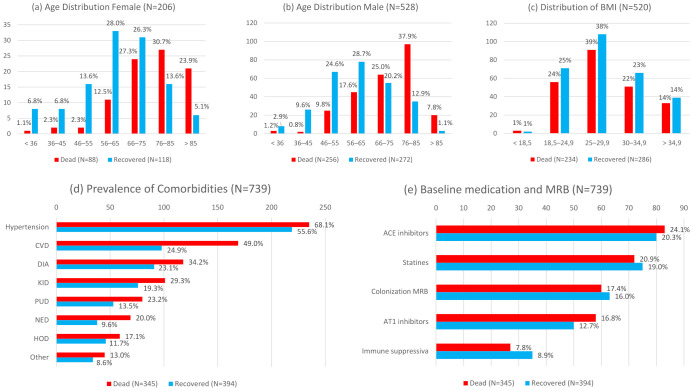
Distribution of baseline values. All axes show absolute frequencies, and percentages are given relative to the subgroup of recovered (blue bars) and dead patients (red bars). Of N = 739 patients included in the study, 394 (53.3%) recovered and 345 (46.7%) died. (**a**) Age distribution of female patients. Overall, 88 of 206 (42.7%) female patients died. (**b**) Age distribution of male patients. Overall, 256 of 528 (48.5%) male patients died. For both genders, the percentage of fatal outcomes increased with age. (**c**) Distribution of the body mass index (BMI) of N=520 patients. Data from remaining patients were missing. Three quarter of the patients were overweight or obese. The distribution of BMI for recovered and dead patients was practically identical. (**d**) Prevalence of comorbidities. Hypertension was the most common comorbidity, both in recovered and dead patients. The largest difference between recovered and dead patients was observed for cardiovascular disease. Generally, all prevalences are higher for patients who died than for patients who recovered. (**e**) Baseline medication and colonization with multi-resistant bacteria (MRB). ACE inhibitors was the most frequently used class of medication observed at baseline for recovered and dead patients, followed by statins. Generally, the differences in medication and known colonization with MRB between dead and recovered patients were small.

Figure 1 contains an overview of all 16 factors measured at baseline, stratified by survival. Of the 739 critically ill COVID-19 patients in this study, 528 (71.4%) were males, while 206 (28.6%) were females. The distribution of age by gender (Figure 1a,b) shows a growing proportion of fatal outcomes with increasing age, both for males and females. We observed 88 of 206 (42.7%) fatal outcomes for female patients, compared to 256 of 528 (48.5%) male patients. The distribution of BMI (Figure 1c) looks almost identical for recovered and for dead patients. In both groups, one quarter of all patients have a BMI in the normal range (BMI 18.5–25), while 38–39% are overweight (BMI 25–30) and 36–37% are obese (BMI above 30).

Hypertension is by far the most common comorbidity (Figure 1d), both in recovered (R) (219 or 55.6%) and dead (D) patients (235 or 68.1%), followed by cardio-vascular disease (R: 98 (24.9%), D: 169 (49%)), diabetes (R: 91 (23.1%), D: 118 (34.2%)), and kidney diseases (R: 76 (19.3%), D: 101 (29.3%)). All comorbidities are more frequent for patients who died than for recovered patients. The highest increases in relative risk can be found for neurological diseases (108%), cardio-vascular diseases (96%), and pulmonary diseases (71%). In contrast, the distribution of medication at baseline level is very similar for recovered and dead patients (Figure 1e). Most frequently patients took ACE inhibitors (R: 80 (20.3%), D: 83 (24.1%)), followed by statins (R: 75 (19.0%), D: 72 (20.2%)), AT1 inhibitors (R: 50 (12.7%), D: 58 (16.8%)), and immune suppresiva (R: 35 (8.9%), D: 27 (7.8%)). A colonization with a multi-resistent pathogen was known for 63 (16.0%) of the recovered and 60 (17.4%) of the dead patients.

### 3.2. Logistic Regression Model and Odds Ratios

The lasso regression selected 15 factors that were influential for the estimation of the risk of death. These factors were used to build a logistic regression model. Only one factor (hemoglobin) had to be removed from the model due to insignificance. Among the remaining 14 variables, 5 were baseline values, while 9 were observed during the critical phase of the disease. The final logistic model was validated with 1000 bootstrap samples, resulting in an area under the receiver operating characteristic (AUROC) of 0.88 (95% CI: 0.86–0.91).

The estimates and odds ratios of the remaining 14 independent factors contributing to risk of death from COVID-19 for critically ill patients are displayed in Table 3. These factors contain 5 out of the original 16 baseline values: age (OR 1.08, CI 1.06–1.10), CVD (OR 1.64, CI 1.06–2.55), PUD (OR 1.87, CI 1.16–3.03), and Statins (0.54, CI 0.33–0.87). One vital parameter (SpO2: unit = 1%, OR 0.94, CI 0.92–0.96) and four blood values are represented in the model: leukocytes (unit 1000/μL, OR 1.04, CI 1.01–1.07), lymphocytes (unit 100/μL, OR 0.96, CI 0.94–0.99), platelets (unit 100,000/μL, OR 0.70, CI 0.62–0.80), and PCT (unit ng/mL, OR 1.11, CI 1.05–1.18). Finally, four symptoms that occurred during the critical phase increased the risk of death are included: kidney failure, expressed as need of dialysis while on ICU (OR 1.68, CI 1.05–2.70), congestive heart failure (OR 2.62, CI 1.11–6.21), severe liver failure (OR 4.93, CI 1.94–12.52), and a quick SOFA score of 3 (OR 1.78, CI 1.14–2.78).

### 3.3. Nomogram

**Figure 2:**
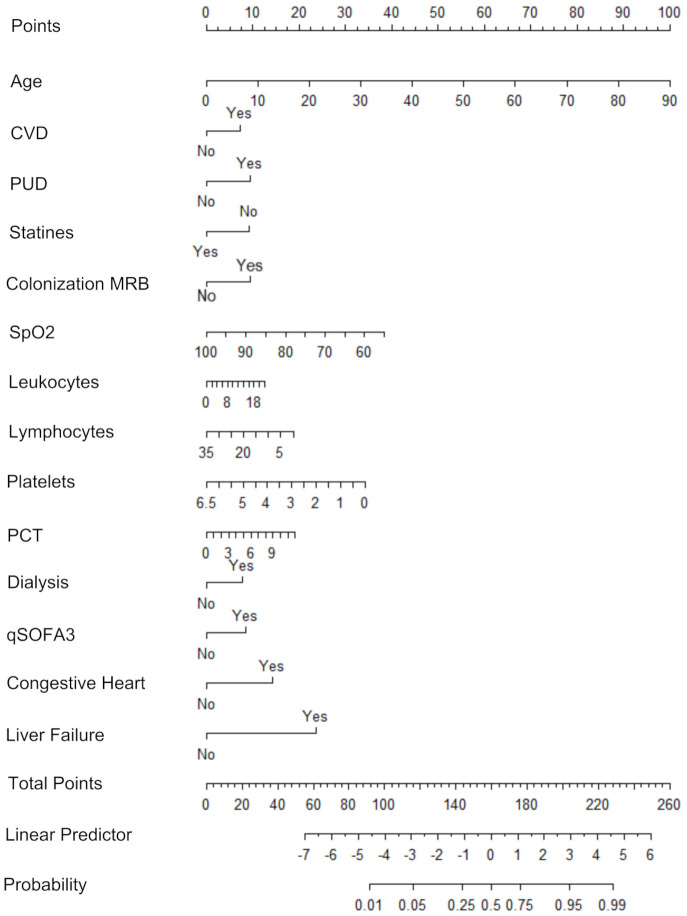
Nomogram graphically representing the logistic regression model for the probability of death of critically ill COVID-19 patients. The length of the scale next to each factor represents the relative importance of the corresponding variable. For a graphical estimation of the probability, first points corresponding to each factor can be found by a vertical line to the top scale. Adding all points results in the total points for this patient. Drawing a vertical line from the total points to the probability scale (scale at bottom) estimates the probability for a fatal outcome.

Based on the final logistic regression model, we built a nomogram for an easy graphical representation of the risk factors and the model (Figure 2). In a nomogram, each factor is represented by a horizontal line with an individual scale for this factor. Comparing a unit change or the length of the line for binary factors allows one to quickly assess the relative importance of the corresponding variable. For example, a known colonization with a multi-resistant pathogen and a pulmonary disease contribute about the same to the total mortality risk, while a liver failure during the critical phase is about twice as relevant for the risk of death.

Historically, nomograms were used for a quick graphical estimation of the probability of the response variable. To this end, each factor is transformed into a number of points at the top of the diagram by drawing a vertical line from the corresponding level. The points are then added to form a total sum. On one hand, the total points can be interpreted as a continuous risk score for mortality. On the other hand, the total points can be converted into a probability of death by drawing a vertical line from the total points scale to the probability scale at the bottom of the diagram.

## 4. Discussion

The COVID-19 pandemic has put a serious burden on healthcare and political systems worldwide. As of June 2021, there have been 172,242,495 confirmed cases of COVID-19, including 3,709,397 deaths reported by the World Health Organisation [1].

In this study, we developed a statistical model for fatal outcome in critically ill COVID-19 patients done by a multivariate analysis that is not based on the classical univariate selection for inclusion of parameters, but on a multifactorial logistic lasso regression for the binary response survival, adding statistical accuracy to a commonly applied model. The model is based on 14 different parameters that had shown to be independent risk factors for mortality in severe courses of COVID-19. The validation of the model using bootstrap samples yielded an AUROC of 0.88. This shows a surprisingly high discriminative power, considering the complexity of the underlying disease. The individual importance of the risk factors can be estimated graphically with the help of the given nomogram.

With our data, we could confirm previously described risk factors for severe COVID-19 such as male gender (71.7%), obesity (30.7%) and hypertension (65.0%) resulting in hospital admission of 739 adult patients across 78 large Hospital Centers in Germany and across Europe. We also reported that the overall mortality of critically ill COVID-19, most of them treated on ICUs, remained high, with 46.7% in our population matching data observed in Europe and the US.

In our model we focus on estimating the probability of survival for hospitalized patients in the critical phase of COVID-19. Variables that seem to weigh high and lead to a rapid increase in mortality were advanced age, low oxygen saturation, and liver failure, combined responsible for up to 75% probability of death. When adding common laboratory findings in severe COVID-19 such as lymphocytopenia and thrombocytopenia, the probability of death can increase to more than 90%. To date, most COVID-19 scores focus on outcome prediction for patients upon hospital admission. Our model is the first one to combine demographics, and clinical and laboratory parameters into a comprehensive probability score for critically ill COVID-19 patients.

A powerful risk factor for mortality from COVID-19 in our study is older age, independent of gender. A unit odds ratio of 1.08 corresponds to doubling the relative risk for every increase of 9 years. Recent studies are in keeping with older age being a risk factor for ICU mortality in COVID-19. Petitt et al. described older age as being an independent risk factor for needing ventilator support in the ICU as well as an independent risk factor for overall mortality [44].

A meta-analysis by Lim et al. found a strong correlation between age and case-fatality-rates in ventilated ICU-patients. Forty-five percent of all ventilated COVID-19 patients died. More than 80% of ventilated ICU patients died when they were 80 years or older [45].

Medical treatment options of critically ill patients with COVID-19 have been rapidly evolving. Although there have been promising therapies such as remdesivir [46] and dexamethasone [47], supportive treatment often requiring ventilator support or at least the application of high doses of oxygen continues to be the mainstay of management of severe COVID-19.

ACE-inhibitors were the most frequently used class of medication in our patient population immediately followed by statins. As for ACE-inhibitors, no difference in outcome could be observed in our patient collective, which is in keeping with the current literature. Over the course of the pandemic, the role of ACE-raising drugs and their clinical implications on COVID-19 have been extensively studied. At this point, the use of ACE-inhibiting drugs seems to be safe in COVID-19, and even though most patients suffering from COVID-19 take ACE-inhibitors this seems to be a confounding factor, as the underlying disease that these medications are prescribed for such as hypertension play a far bigger role for adverse outcomes in COVID-19 [48].

However, the use of statins prior to admission seemed to exhibit protective effects in our patient population and is one of the 14 parameters for the logistic regression model. Our finding is in keeping with a recent American study [49], showing a reduced risk of a severe progression of COVID-19. The statins factor also clearly demonstrates the necessity of careful statistical methods. Univariately, statins have a *p*-value of 25.9% in our data set and would therefore never have been considered if the variable selection process was falsely based on univariate analysis.

The use of statins as a treatment option in COVID-19 is controversial. However, the recent meta-analysis of Kow and Hasan of four major studies including more than 8900 patients showed a reduction in fatal or severe disease by 30% when statins were used compared to non-use of statins [50]. Currently, two opposing views on the effects of statins on the clinical course of COVID-19 are being discussed in the literature. On the one hand, researchers postulate that statins could protect COVID-19 patients from the development of overwhelming inflammatory responses [51]. On the other hand, statins might be protective towards lung injury by upregulation of ACE-expression [52]. Large randomized trials are missing to date but are needed to clarify this issue.

Many risk factors for a severe course of disease as well as fatal outcomes in COVID-19 have been studied, and some have been identified [45]. A few comorbidities have emerged as major risk factors for hospitalization and ICU admission as well as death from COVID-19 since the beginning of the pandemic. Early on, data from China could identify hypertension, diabetes, chronic pulmonary disease, and cardiovascular disease as contributing factors for a severe course. These comorbidities could then be confirmed all over the world. Further studies then showed obesity and the as male gender to be significant risk factors for hospital admission and a severe course of disease [44].

One of the most striking findings of our study is that commonly accepted risk factors for COVID-19 mortality such as male gender and obesity cannot be confirmed in our final model. This means that once COVID-19 patients are critically ill, typically requiring ICU care, gender and increased BMI do no longer represent independent risk factors for increased mortality. Even though only one quarter of our patients had a BMI in the normal range (18.5–25) and the majority of patients were overweight or obese, the distribution of BMI for recovered and dead patients was practically identical. The same phenomenon accounts for gender. Although 72.2% of our patients were males, no difference could be found between mortality in males versus females in our population: overall, 88 of 206 (42.7%) female patients and 256 of 528 (48.5%) male patients died (42.7%) once they were critically ill.

With regard to comorbidities, we obtained a similar result. Our baseline factors, cardiovascular disease (CVD), chronic pulmonary disease (PUD), diabetes, and hypertension, are well-established risk factors for COVID-19 in the general population [53]. CVD and PUD are significant independent factors in our model; hence, they still contribute to the risk of death once patients are critically ill. However, in our data set, this is not the case for diabetes and hypertension.

At this point it remains unclear as to why gender, BMI, diabetes and hypertension do not represent significant risk factors for adverse outcome in critically ill patients. However, our study is the first one to use the lasso method for logistic regression and for generating independent risk factors exclusively for critically ill patients in that matter. Most studies use patient populations composed of all symptomatic patients or all hospitalized patients, not distinguishing between ICU mortality and non-ICU mortality. Regarding BMI, it should be noted that our original data set contained 29.6% missing values before imputation. In addition, the lasso variable selection mechanism could have been too parsimonious, as it excludes variables in case of strong correlation or multicollinearity. Further studies with larger patient numbers will be needed to confirm these findings regarding gender, obesity, BMI, and comorbidities.

The factors in our model confirm that severe COVID-19 always has to be considered as a multi-organ disease, as already described in the early phase of the pandemic [54,55]. Therefore, special attention should be paid to survivors with bad prognosis due to old age and multimorbidity [56].

Our study could serve as a starting point for the development of a risk score that is specific to critically ill COVID-19 patients. Once such a score has been validated, it would be of high interest to compare its performance with scores for COVID-19 developed by others [36,57,58], and with classic ICU mortality scores such as SAPS, SOFA, or NEWS2 [59].

Our study has limitations. Being multicentric and part of an evolving system over the course of the pandemic, centers were continuously added to the LEOSS data bank. Considering the large number of contributing centers, some degree of heterogeneity in the data collection protocol had to be accepted. The exclusion of 101 patients (12.0%) due to their last known status could potentially introduce bias. Another potential source of bias lies in the fact that few critically ill patients refuse invasive treatment on ICUs. In addition, most patients were Germans treated in German hospitals. Therefore, generalisability to other countries with, for instance, a different distribution of socio-economic status or ethnicity might be limited. In the original data set, we encountered a significant proportion of missing data. We excluded variables with too many missing values and used data imputation for the remaining ones, but this typically limits the predictive power of the resulting models. In addition, all factors in the data set were categorized at the time of data collection. This can lead to reduced precision, over- or underestimation of effects, and reduced power. Finally, our model was generated post hoc, so that one can expect a certain loss of accuracy when our results are generalized to other patient samples. In summary, all results should be externally validated on independent test sets.

## 5. Conclusions

Our study identified specific risk factors for critically ill COVID-19 patients that differ from known risk factors for the general population. The 14 parameters in our model—demographics, clinical parameters, and some common laboratory findings—can help clinicians gain a better understanding for the subgroup of patients who are at greatest risk of dying from severe courses of COVID-19.

## Figures and Tables

**Table 1:** Comorbidities formed by aggregating groups of diseases. A comorbidity was recorded if at least one specific disease was indicated.

Comorbidity	Disease
hypertension	hypertension
cardiovascular disease	myocardial infarction
(CVD)	atrioventricular block
	carotid arterial disease
	chronic heart and circulation failure
	peripheral vascular disease
	atrial fibrillation
	coronary artery disease
pulmonary disease	COPD,
(PUD)	other chronic pulmonary diseases
hematological/oncological disease	leukemia
(HOD)	lymphoma
	solid tumor
	stem cell transplantation
diabetes mellitus	diabetes without organ damage
(DIA)	diabetes with organ damage
kidney disease	acute kidney injury
(KID)	chronic kidney injury
neurological diseases	hemiplegia
(NED)	dementia
	cerebrovascular disease
	stroke
other diseases	peptic ulcer disease
(OtherD)	chronic liver disease
	liver cirrhosis
	organ transplantation
	rheumatic disease
	HIV/AIDS

**Table 2:** Variables measured during the critical phase of the COVID-19 disease. Bold typeface indicates that the variable is an independent risk factor in our logistic regression model. PaO2 = partial pressure of oxygen (mmHg).

Group	Factor
vital parameters (6)	systolic blood pressure, diastolic blood pressure, pulse temperature, respiratory rate, **pulse oxygen saturation (SpO2)**
blood values (8)	D-dimer, **leukocytes**, **lymphocytes** neutrophiles, **platelets**, hemoglobin, CRP (C-reactive protein), **PCT (procalcitonin)**
symptoms (8)	**ICU dialysis**, fungal superinfection, septic shock, **quick SOFA of 3**, **congestive heart failure**, life threatening cardiac arrhyhtmia, PaO2 below 60, **severe liver failure**
CT results (4)	areas of consolidation, crazy paving, ground glass, pleural effusion

**Table 3:** Results of the multifactorial logistic regression for the binary response survival (dead versus recovered). The bootstrap validation of the model yielded an AUROC of 0.88 (95% CI: 0.86; 0.91). The columns show the parameter estimate, the *p*-value, and the odds ratio (OR) with its 95% confidence level. For continuous variables, the unit is indicated. For all other variables, the odds ratio refers to the occurrence of the specified event versus non-occurrence.

Factor	Estimate	*p*-Value	OR	Lower 95%	Upper 95%
Intercept	−0.1913	0.8630			
Baseline values					
Age (1 year)	0.0746	<0.0001	1.08	1.06	1.10
CVD	0.4955	0.0276	1.64	1.06	2.55
PUD	0.6304	0.0098	1.87	1.16	3.03
Statins	−0.6179	0.0118	0.54	0.33	0.87
Known Colonization	0.6391	0.0142	1.89	1.14	3.16
Lab values and vitals (changes per unit)					
Leukocytes (1000/μL)	0.0384	0.0206	1.04	1.01	1.07
Lymphocytes (100/μL)	−0.0359	0.0208	0.96	0.94	0.99
Platelets (100,000/μL)	−0.3537	<0.0001	0.70	0.62	0.80
PCT (ng/mL)	0.1071	0.0001	1.11	1.05	1.18
SpO2 (1%)	−0.0572	<0.0001	0.94	0.92	0.96
Symptoms					
Dialysis on ICU (Kidney Failure)	0.5198	0.0318	1.68	1.05	2.70
Congestive Heart Failure	0.9629	0.0287	2.62	1.11	6.21
Severe Liver Failure	1.5953	0.0008	4.93	1.94	12.52
qSOFA = 3	0.5745	0.0117	1.78	1.14	2.78

## Data Availability

Patient data from the LEOSS registry are subject to the LEOSS governance, data use, and access policy (policy text available on https://leoss.net, accessed on 16 July 2021).
